# Kidney mitochondrial DNA contributes to systemic IL-6 release in sepsis-associated acute kidney injury

**DOI:** 10.1172/jci.insight.177004

**Published:** 2025-12-08

**Authors:** Avnee J. Kumar, Katharine Epler, Jing Wang, Alice Shen, Negin Samandari, Mark L. Rolfsen, Laura A. Barnes, Gerald S. Shadel, Alexandra G. Moyzis, Alva G. Sainz, Karlen Ulubabyan, Kefeng Li, Kristen Jepsen, Xinrui Li, Mark M. Fuster, Roger G. Spragg, Roman Sasik, Volker Vallon, Helen Goodluck, Joachim H. Ix, Prabhleen Singh, Mark L. Hepokoski

**Affiliations:** 1 VA San Diego Healthcare System, San Diego, California, USA.; 2 Division of Pulmonary and Critical Care and Sleep Medicine, UCSD, La Jolla, California, USA.; 3 Department of Critical Care Medicine, Yantai Yuhuangding Hospital, Affiliated with Medical College of Qingdao University, Yantai, Shandong, China.; 4 Department of Medicine, School of Medicine, UCSD, La Jolla, California, USA.; 5 Vanderbilt University, Nashville, Tennessee, USA.; 6 Salk Institute for Biological Sciences, La Jolla, California, USA.; 7 Department of Pathology, Yale School of Medicine, New Haven, Connecticut, USA.; 8 Faculty of Applied Sciences, Macao Polytechnic University, Macao, China.; 9 Institute for Genomic Medicine (IGM),; 10 Center for Computational Biology & Bioinformatics, and; 11 Division of Nephrology and Hypertension, UCSD, La Jolla, California, USA.

**Keywords:** Inflammation, Nephrology, Innate immunity, Mitochondria

## Abstract

Mitochondrial dysfunction is a major mechanism of acute kidney injury (AKI), and increased circulating interleukin 6 (IL-6) is associated with systemic inflammation and death due to sepsis. We tested whether kidney mitochondrial DNA (mtDNA) contributes to IL-6 release in sepsis-associated AKI via Toll-like receptor 9 (TLR9). In a murine model of sepsis via cecal ligation and puncture (CLP), we used next-generation sequencing of plasma mtDNA to inform the design of optimal target sequences for quantification by droplet digital PCR, and to identify single-nucleotide polymorphisms (SNPs) to infer tissue origin. We found significantly higher concentrations of plasma mtDNA after CLP versus shams and that plasma mtDNA SNPs matched kidney SNPs more than other organs. Kidney mtDNA contributed directly to IL-6 and mtDNA release from dendritic cells in vitro and kidney mitochondria solution led to higher IL-6 concentrations in vivo. IL-6 release was mitigated by a TLR9 inhibitor. Finally, plasma mtDNA was significantly higher in septic patients with AKI compared with those without AKI and correlated significantly with plasma IL-6. We conclude that AKI contributes to increased circulating IL-6 in sepsis via mtDNA release. Targeting kidney mitochondria and mtDNA release are potential translational avenues to decrease mortality from sepsis-associated AKI.

## Abstract 2

Mitochondrial DNA released from the kidney contributes directly to interleukin-6 release during sepsis associated-acute kidney injury

## Introduction

Sepsis is a syndrome characterized by a dysregulated innate immune response to an infection and causes 11 million deaths annually throughout the world (1, 2). Death due to sepsis most often occurs as part of multiple-organ dysfunction syndrome (MODS). Acute kidney injury (AKI) in sepsis has been shown to carry a greater risk of mortality compared with most other organ failures (3), and sepsis is the most common cause of AKI in the critically ill (4). The mechanisms involved in increased mortality in sepsis-associated AKI (S-AKI) are not well understood, but recent data have demonstrated that AKI is a systemic disease that may contribute directly to MODS (5–9).

Systemic mitochondrial dysfunction is a key pathophysiological feature of sepsis (10–12), and mitochondrial damage and dysfunction are well-established mechanisms of various forms of AKI (7), including S-AKI (13). Mitochondrial dysfunction not only leads to metabolic disruptions but can also lead to release of mitochondrial damage-associated molecular patterns (mtDAMPs) that activate innate inflammatory and apoptotic pathways (14–16). Mitochondrial DNA (mtDNA) is one such mtDAMP that has been heavily implicated in the pathogenesis of sepsis, and higher circulating, cell-free mtDNA (CCF-mtDNA) levels are associated with mortality in septic patients (17–21). Like bacterial DNA, mtDNA contains cytosine-phosphate-guanine (CpG) motifs that are hypo- or unmethylated (22) and thus contribute to the systemic inflammatory response by acting as strong agonists of Toll-like receptor 9 (TLR9) (23). The exact source of the elevated plasma mtDNA concentrations in sepsis has not been clearly established. Interestingly, one study showed the copy number of mtDNA in the kidney decreased acutely in S-AKI, suggesting mtDNA was released from kidney cells during sepsis (24).

Investigations into the role of kidney mtDNA in sepsis have yielded conflicting results (25, 26), which may be due to several factors. First, the size and sequence of CCF-mtDNA fragments in sepsis have not been well characterized; therefore, methods to quantify mtDNA by quantitative PCR (qPCR) have been highly variable and lack reproducibility (19, 27). Furthermore, most mtDNA genes are also present in the nucleus as pseudogenes (28), often called nuclear-mitochondrial segments (NUMTs). Therefore, qPCR probes that do not avoid NUMTs may yield false-positive results. Finally, mitochondrial damage develops in the early phases of AKI (29), and kidney mtDNA may be released and cleared from the circulation before clinical AKI is recognized.

We hypothesized that AKI contributes directly to increased concentrations of plasma CCF-mtDNA in sepsis, and that kidney mtDNA contributes to systemic IL-6 release via TLR9. To test these hypotheses, we performed next-generation sequencing (NGS) of CCF-mtDNA from the plasma of mice exposed to sepsis via cecal ligation and puncture (CLP) to gain insights into the size and sequence of CCF-mtDNA fragments. We utilized these data and a NUMT analysis to optimize mtDNA quantification in mice by droplet digital PCR (ddPCR), congruent with similar work we have demonstrated in humans (30). To infer tissue origin of CCF-mtDNA, we used our NGS data to identify plasma mtDNA heteroplasmy (mixtures of mtDNA genotypes due to single-nucleotide polymorphisms [SNPs]) after CLP and compared plasma mtDNA SNPs to those found in the heart, kidney, liver, buffy coat, and lung from individual mice. Circulating levels of mtDNA in the critically ill are also associated with specific metabolic changes (31); thus, we compared the metabolome of the kidney of mice following CLP to these same organs. Finally, we investigated the potential for kidney mtDNA to cause IL-6 release in vitro and in vivo and compared CCF-mtDNA concentrations in septic patients with and without AKI.

## Results

### Systemic cytokines are increased prior to elevations in plasma creatinine after CLP.

**Figure 1:**
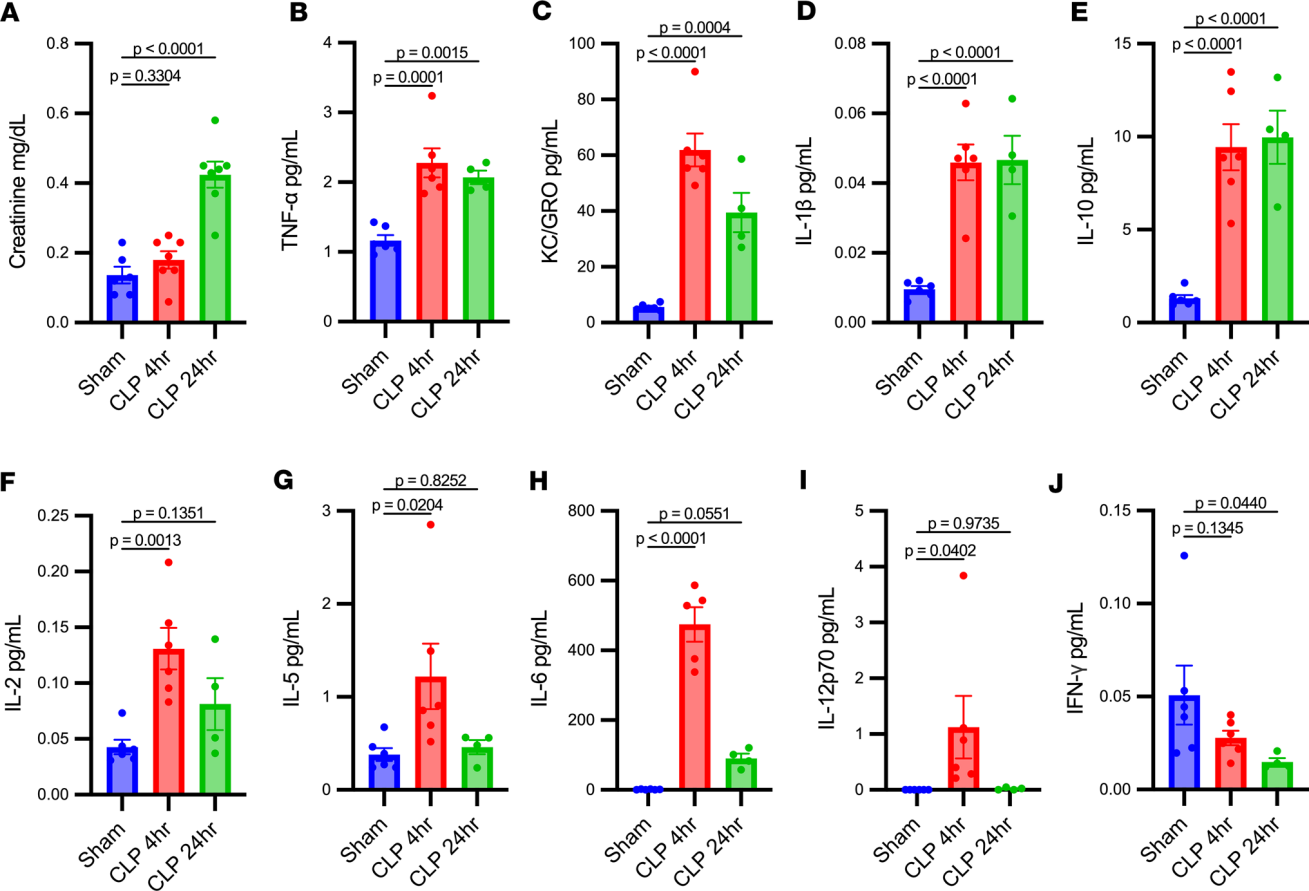
Systemic cytokines are increased prior to plasma creatinine in cecal ligation and puncture models of sepsis-associated AKI. Plasma creatinine (**A**) was significantly elevated 24 hours after cecal ligation and puncture (CLP) compared with sham operation, but no significant differences were found at the 4-hour time point. Conversely, TNF-α (**B**), KC/GRO (**C**), IL-1β (**D**), IL-10 (**E**) increased and remained increased compared with sham at 24 hours and 4 hours. IL-2 (**F**), IL-5 (**G**), IL-6 (**H**), and IL-12p70 (**I**) increased at 4 hours, but returned to sham levels at 24 hours. Plasma levels of IFN-γ (**J**) decreased at 24 hours in CLP compared with sham. Significant differences between groups were determined by 1-way ANOVA with Fisher’s LSD post hoc analysis. **A**, *n* = 6–7/group; **B**–**J**, *n* = 4–6/group.

Plasma creatinine and levels of inflammatory cytokines were evaluated after CLP at 4- and 24-hour time points (Figure 1). Plasma creatinine was significantly increased after CLP compared with sham operation at 24 hours, but no significant differences were found at the 4-hour time point (Figure 1A). Plasma tumor necrosis factor α (TNF-α), keratinocyte chemoattractant/human growth-regulated oncogene (KC/GRO), IL-1β, IL-10, IL-2, IL-5, IL-6, and IL-12p70 increased significantly at 4 hours compared with sham-operated mice. Plasma TNF-α, KC/GRO, IL-1β, and IL-10 remained significantly elevated at 24 hours compared with sham mice, while IL-2, IL-5, IL-6, and IL-12p70 did not (Figure 1, B–I). Plasma IFN-γ (Figure 1J) did not significantly differ between CLP and sham mice at 4 hours and decreased at 24 hours. Plasma IL-4 (data not shown) did not significantly differ between CLP and sham mice at either time point. The change in concentration between CLP and sham mice was greatest for IL-6 compared with all other cytokines.

### Total CCF DNA and mtDNA are highly fragmented after CLP.

**Figure 2:**
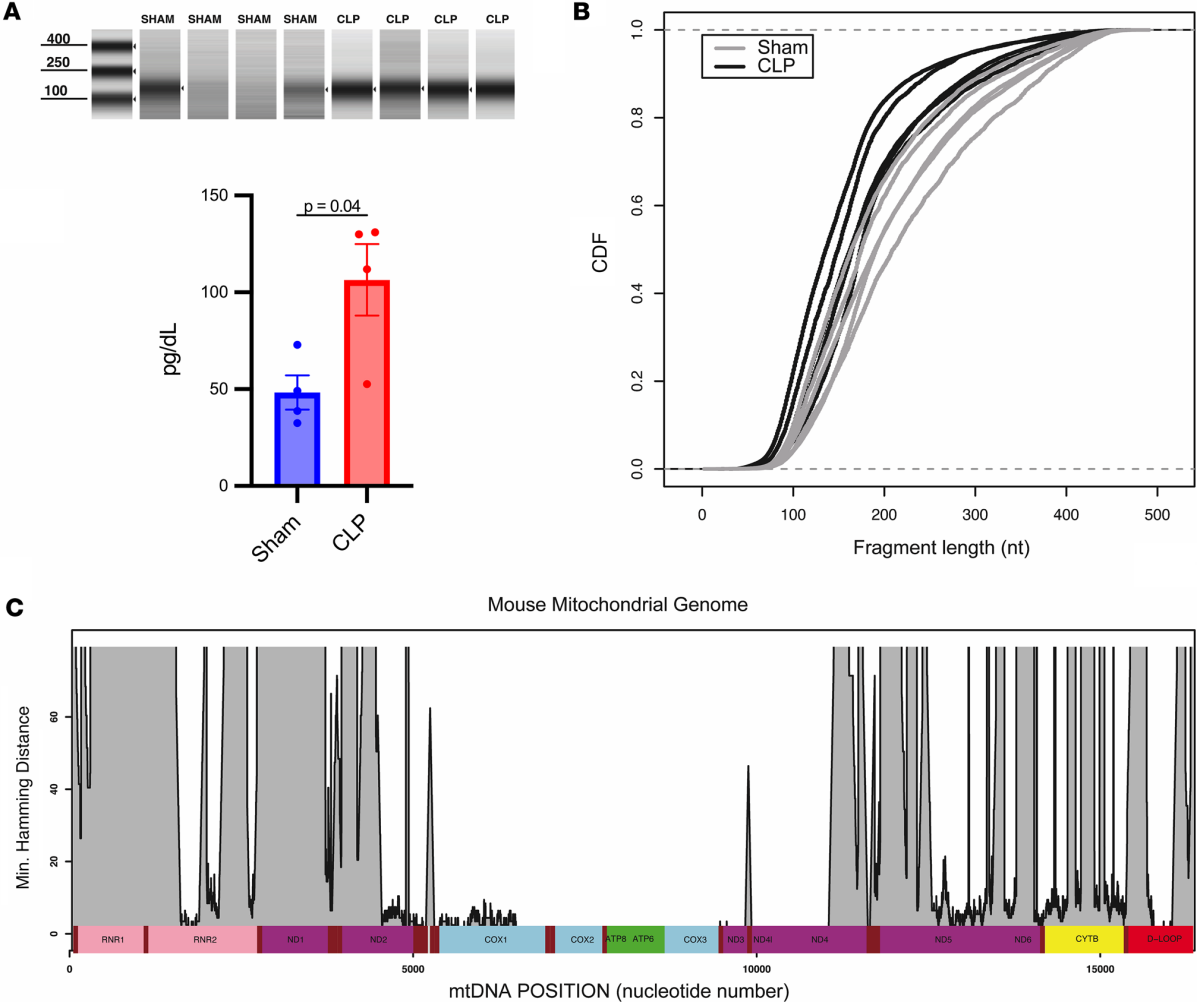
Circulating cell-free DNA fragments are increased after cecal ligation and puncture compared with sham operation and circulating cell-free mtDNA is highly fragmented. DNA was isolated from mouse plasma 4 hours after cecal ligation and puncture (CLP) versus sham operation. Next-generation sequencing was used to determine the size distribution of the circulating cell-free mtDNA fragments. Minimum Hamming distance for the entire mouse mitochondrial genome compared to the nuclear genome was determined by blastn in the ungapped mode. (**A**) The concentrations of total cell-free DNA fragments between 100 and 150 bp in length were determined by automated electrophoresis and found to be increased significantly in CLP mice versus shams. (**B**) Size distribution analysis of circulating cell-free mtDNA fragments showed a high degree of fragmentation after CLP, with roughly 40% of all fragments being less than 150 bp and roughly 15% being less than 100 bp in length. The overall size distribution of circulating cell-free mtDNA fragments did not differ significantly between CLP mice versus shams. (**C**) Several regions of the mouse mitochondrial genome, such as within the cytochrome *c* oxidase (COX) subunits, have very low minimum Hamming distances compared with the nuclear genome, indicating a high degree of similarity to a region of nuclear DNA. Significant differences between groups were determined by unpaired, 2-tailed *t* tests comparing the group means ± SEM. **A**, *n* = 4/group; **B**, *n* = 5/group. CDF, cumulative distribution function.

There was a significant increase in fragmented CCF DNA of 100–150 bp in size in CLP versus sham mice (Figure 2A). NGS provided an mtDNA yield of 0.24%–12% relative to nuclear DNA, with 34,804–1,500,550 reads per sample mapping to the mitochondrial genome available for downstream analyses (https://www.ncbi.nlm.nih.gov/sra/PRJNA899505). Next, the size distribution of mtDNA fragments from CLP versus sham mice showed a trend toward a smaller distribution after CLP injury (Figure 2B). Size distribution analysis also revealed that roughly 10%–20% of sequenced mtDNA fragments were less than 100 bp in length. Minimum Hamming distance (minimum number of mismatches between each mtDNA fragment and the best match within the entire nuclear genome via blastn) was used to evaluate which sequences of the mitochondrial genome have the potential to exist as NUMTs. Figure 2C shows the minimum Hamming distance by location for the entire mouse mitochondrial genome compared to the nuclear genome. Several locations, such as in the cytochrome *c* oxidase subunit 2 (COX2), were found to have minimum Hamming distances of 0, indicating that they have exact matches within the nuclear genome. Other locations, such as within NADH dehydrogenase 1 (ND1) and cytochrome B (CytB), have very high minimum Hamming distances, suggesting they are highly unlikely to exist as NUMTs and hence are optimal regions for ddPCR target sequences with higher specificity for mtDNA.

### Plasma mtDNA concentrations are increased early after CLP.

**Figure 3:**
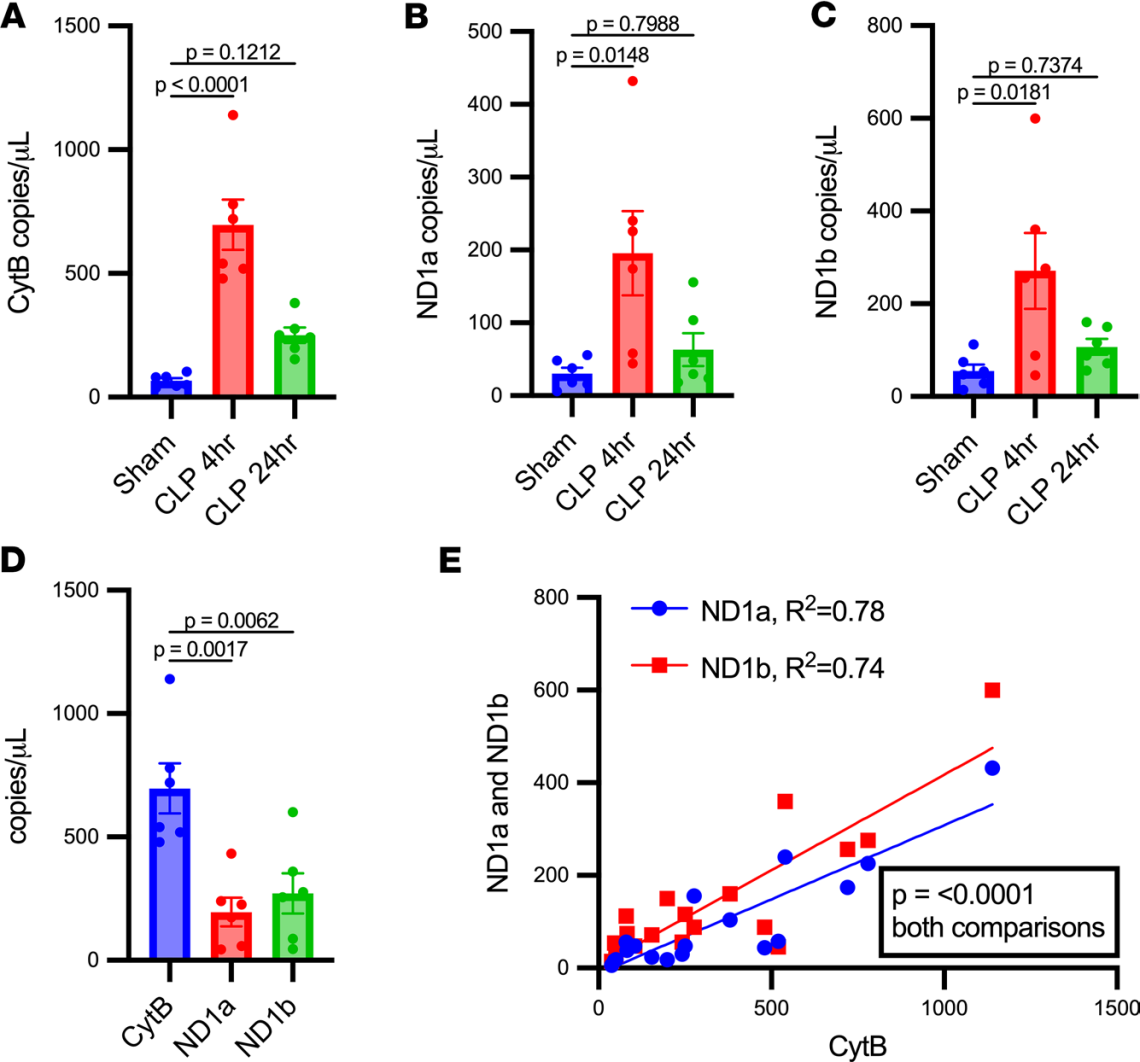
Circulating cell-free mtDNA is increased in mice after cecal ligation and puncture compared with sham operation. Plasma concentrations of mtDNA were determined by droplet digital PCR targeting sequences within the cytochrome *b* (CytB) and NADH dehydrogenase 1 (ND1) genes. These genes were chosen based on their dissimilarity from any region of the nuclear genome based on minimum Hamming distance, and small sequences were targeted to account for fragmentation. Plasma concentrations of CytB (**A**), ND1a (**B**), and ND1b (**C**) were all increased significantly in cecal ligation and puncture (CLP) mice compared with shams at 4 hours. CytB and ND1b remained significantly increased at 24 hours compared with shams, and CytB was also increased significantly at 4 hours versus 24 hours after CLP. (**D**) The smallest target sequence, CytB, was increased significantly compared with ND1a and ND1b at 4 hours after CLP. (**E**) Simple linear regression between plasma concentrations of CytB and ND1a and ND1b, respectively, showed a positive and significant correlation for both comparisons. Significant differences between groups were determined by 1-way ANOVA with Tukey’s post hoc analysis. *n* = 6/group.

Three sets of primer/probes were identified for mice that target small mtDNA sequences in the CytB (93 bp) and ND1 genes (106 and 129 bp for ND1a and ND1b, respectively) (Table 1). The plasma mtDNA concentration determined by all 3 target sequences was significantly increased at 4 hours in CLP compared with sham mice (Figure 3, A–C). At 4 hours, CytB copy number was significantly increased compared with ND1a and ND1b (Figure 3D). However, a simple linear regression of copy number obtained by the CytB versus ND1a and ND1b target sequences showed a positive and significant correlation, indicating they may yield similar results when comparing to biological outcomes (Figure 3E).

### Multiple plasma mtDNA SNPs match SNPs identified in the kidney.

**Figure 4:**
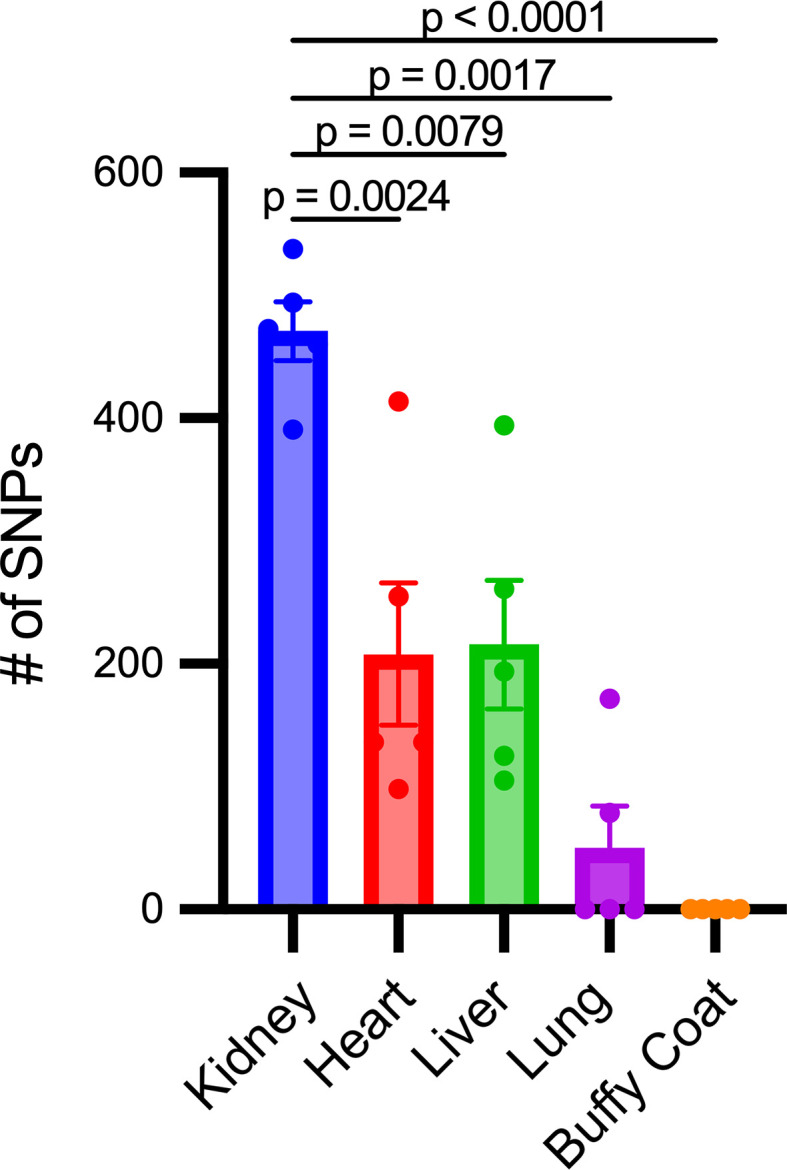
The kidney has increased mtDNA single-nucleotide polymorphisms. When compared with the heart, liver, lung, and buffy coat, the kidney had an absolute increase in single-nucleotide polymorphisms (SNPs) (*P* = 0.0068, *P* = 0.0216, *P* = 0.0049, *P* = 0.001 respectively). Significant differences between groups were determined by 1-way ANOVA with Fisher’s LSD post hoc analysis. *n* = 5/group.

Plasma mtDNA SNPs were identified and compared to those found in the kidney, heart, liver, buffy coat, and lung tissue in mice exposed to CLP to gain insights into the potential tissue origin of CCF-mtDNA. Buffy coat was utilized for evaluation of leukocytes, as prior studies suggest circulating leukocytes are a major source of circulating mtDNA (32). The kidney had significantly more mtDNA SNPs present after CLP compared with the heart, liver, and lung; no SNPs were identified in the buffy coat (Figure 4). Plasma mtDNA SNPs from individual mice that match those found in one tissue site only are shown in Table 2. Four out of 5 mice had at least 1, and up to 8, plasma mtDNA SNPs, that were also present in the kidney, but not in the heart, liver, or lung. Two mice had plasma mtDNA SNPs that were present in plasma and only the lung. The liver and heart each only had a unique SNP matching the plasma in one mouse. Every mouse had at least 1, and up to 4, plasma SNPs that matched multiple organs as demonstrated in Supplemental Table 1 (supplemental material available online with this article; https://doi.org/10.1172/jci.insight.177004DS1), with the kidney being the only organ present in all cases of multiple tissue matches. Finally, each mouse had several plasma mtDNA SNPs that were not present in any of the tissues evaluated (data not shown, but sequencing files available at http://www.ncbi.nlm.nih.gov/bioproject/899505).

### Metabolome comparisons in tissues after CLP.

**Figure 5:**
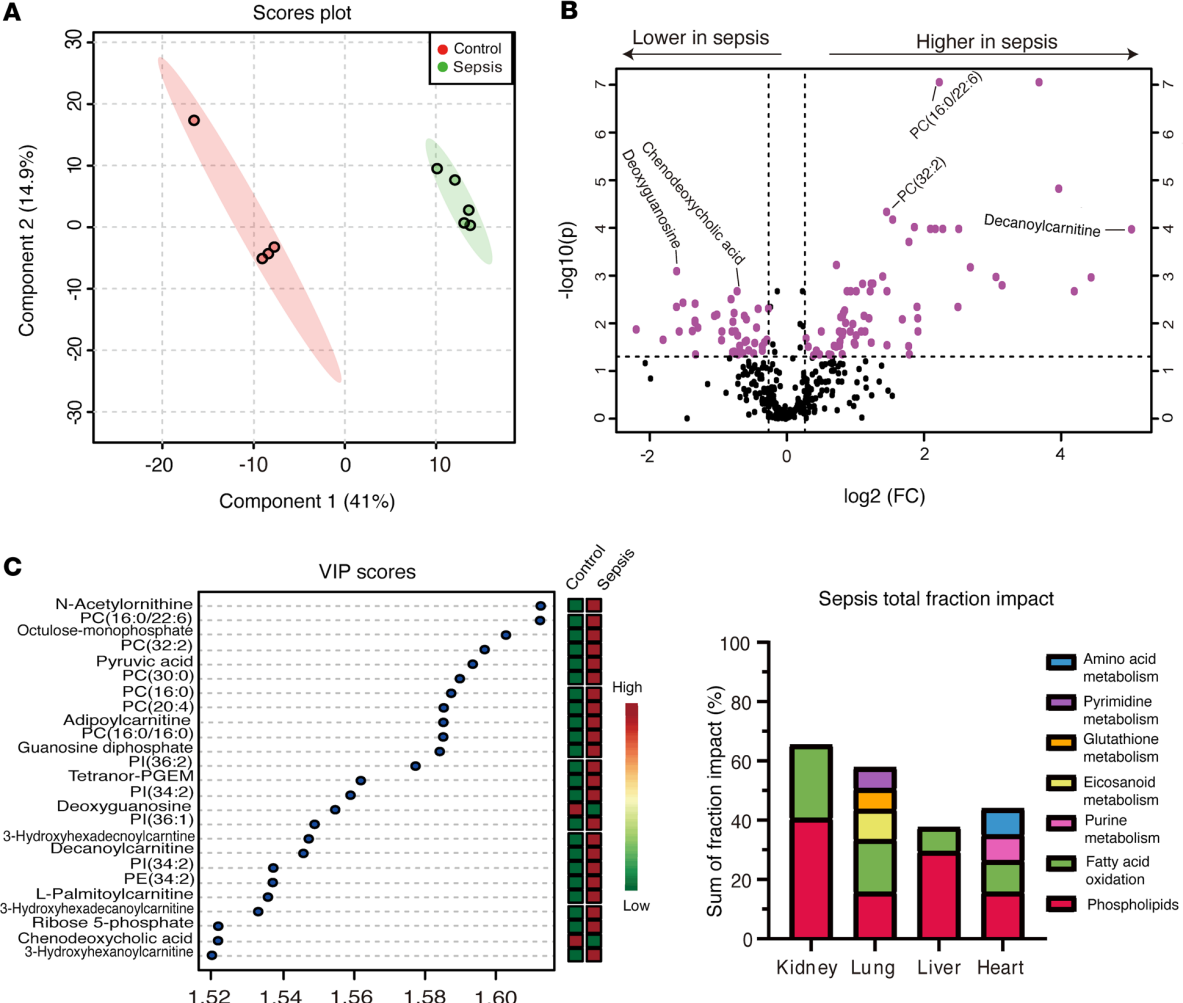
Kidney metabolomic changes are increased compared with other organs after sepsis by cecal ligation and puncture. Next-generation metabolomics was used to evaluate metabolic changes in the kidney after cecal ligation and puncture (CLP) compared to those in the lung, liver, and heart. (**A**) PLS-DA, a supervised multivariate analysis, showing a significant difference in the metabolomic profile in kidney tissue of mice after CLP versus sham operation. (**B**) Volcano plot analysis shows the number of significantly increased or decreased metabolites in kidney tissue of mice after CLP versus sham operation, with 20% of the 483 total metabolites analyzed showing significant differences. Fold change greater than 1.2 and FDR-adjusted *P* value of less than 0.05 were considered significant. (**C**) Top 25 metabolites contributing to metabolomic differences between CLP versus sham groups as ranked by VIP scores showed majority of the most significantly altered metabolites were phospholipids, i.e., phosphatidylcholine [PC(32:2)], or medium- and long-chain acylcarnitines (i.e., adipoylcarnitine). (**D**) Comparison of the sum of the fractional impact of all significantly altered metabolites shows the greatest sum in the kidney followed by the lung, heart, and liver, respectively. *n* = 4 for sham, *n* = 5 for CLP.

Next-generation metabolomics was then utilized to compare the metabolic changes that occur in the kidney after CLP to those found in the heart, liver, and lung. Partial least squares discriminate analysis (PLS-DA), a supervised multivariate analysis, demonstrated a significant separation between the metabolic profile in the kidney of CLP versus sham mice (Figure 5A). A leave-one-out cross-validation (LOOCV) was performed to check the predictive ability of the PLS-DA model, and obtained a Q2 score of 0.84, which implied a high quality PLS-DA model (7). There were also significant metabolomic changes between CLP and sham mice in the heart, liver, and lung, with Q2 scores of 0.6, 0.6, and 0.8, respectively. Next, a volcano plot analysis with a fold-change threshold of 1.2 and adjusted *P* of less than 0.05 was conducted to identify metabolites that were either significantly increased or decreased in the kidney. This analysis showed that over 20% of the 483 total metabolites analyzed were significantly changed in CLP versus sham mice (Figure 5B). The significantly changed metabolites between CLP and sham mice were then ranked by their variable importance in projection (VIP) scores to identify the metabolites and metabolic pathways that were most impacted. Out of the top 25 metabolites most significantly altered in the kidney, 11 were phospholipids, such as phosphatidylcholine — PC(16:0/22:6) and PC(32:2) — and 6 were medium and long-chain acylcarnitines from the fatty acid oxidation (FAO) pathway such as adipoylcarnitine and decanoylcarnitine (Figure 5C). The fold change was greatest for the FAO metabolites, which were increased greater than 3-fold in CLP versus sham mice. Deoxyguanosine, 1 of the 4 deoxyribonucleosides, and chenodeoxycholic acid, a bile acid, were the only metabolites in the top 25 that were significantly decreased in CLP versus sham mice. Finally, the sum of the fractional impact of all the significantly altered metabolites in kidney, heart, liver, and lung tissue from CLP versus sham mice were compared to determine which tissue experienced the greatest metabolomic change. The sum of the fractional impact was highest in the kidney compared with the other organs evaluated (Figure 5D).

### Kidney mtDNA causes IL-6 release from dendritic cells and plasma IL-6 is increased after kidney mtDAMP injection.

**Figure 6:**
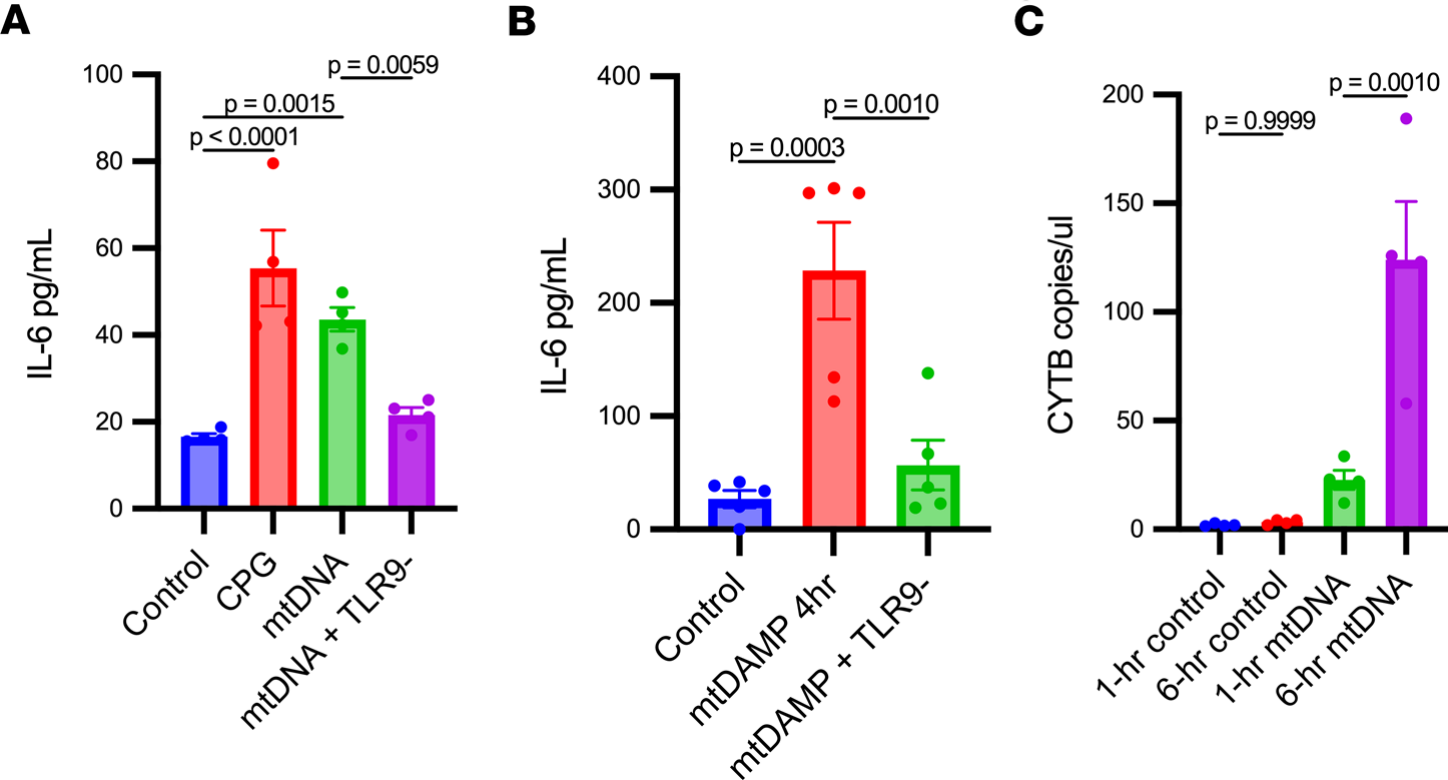
CpG in vitro in DCs and kidney mtDAMP in vivo contributes to IL-6 release. (**A**) DCs isolated from mouse bone marrow were treated with CpG (1 μM) as a positive control or kidney mtDNA (10 μg/mL) and incubated for 4 hours prior to quantification of IL-6 in the culture supernatant. Additional cells were additionally treated with TLR9 inhibitor. Cells treated with CpG and mtDNA had a significant increase in IL-6 in supernatant compared to controls. However, IL-6 concentrations in supernatants from cells treated with mtDNA and a TLR9 inhibitor decreased significantly from mtDNA treatment alone. (**B**) Mice were treated with mtDAMPs followed by measurement of plasma IL-6 at 4 hours with and without TLR9 inhibition. Plasma IL-6 concentrations were increased significantly in mice at 4 hours following subcutaneous injection of mtDAMPs vs normal saline (control). Plasma IL-6 concentrations decreased significantly after mtDNA plus TLR9 inhibition compared mtDNA alone. (**C**) mtDNA levels were measurable via *CYTB*. Significant differences between groups were determined by 1-way ANOVA with Fisher’s LSD (**A** and **B**) or Tukey’s (**C**) post hoc analysis. Experiment in **A** was completed 3 separate times. **A**, *n* = 5 wells/group; **B**, *n* = 4/group.

Next, the impact of mtDNA with and without TLR9 inhibition on IL-6 release from dendritic cells (DCs) isolated from mouse bone marrow in vitro. We chose to focus on IL-6 since it showed the greatest absolute increase compared with other cytokines, and IL-6 is a well-established mediator of systemic inflammation that predicts mortality in the CLP model of sepsis (33). However, we had a comprehensive evaluation of additional cytokines included in our panel as well. For the in vitro experiments, DCs were plated and treated with CpG (1 μM) as a positive control or kidney (10 μg/mL) and incubated for 4 hours prior to quantification of cytokines in the cell culture supernatant. The CpG- and mtDNA-treated cells had a significant increase in IL-6 in the supernatant compared with untreated cells, which was not found with the addition of a TLR9 inhibitor (Figure 6A). Concentrations of TNF-α, KC/GRO, and IL-4 in the supernatant were also increased significantly by mtDNA treatment, which was mitigated by TLR9 inhibition in only TNF-α and KC/GRO. No other cytokines evaluated were significantly increased by mtDNA (Supplemental Figure 1). In vivo, mice were injected with kidney mtDAMP solution to mimic the concentration of mtDNA released during sepsis (15). Mice treated with kidney mtDAMPs had a significant increase in plasma IL-6 at 4 hours compared with mice treated with normal saline, and the plasma IL-6 increase was mitigated by TLR9 inhibition (Figure 6B). Plasma concentrations of IL-10 and IL-2 were also increased by mtDAMP injection, but their concentration was not significantly suppressed by treatment with the TLR9 inhibitor (Supplemental Figure 2). Finally, to test whether kidney mtDNA would lead to mtDNA release from DCs, CytB was compared in the supernatant at 1 and 6 hours after mtDNA treatment. The CytB concentration increased significantly at 6 hours compared with 1 hour after kidney mtDNA exposure, which was not found in controls (Figure 6C).

### Increased plasma mtDNA is associated with S-AKI and correlates with plasma IL-6 in critically ill individuals with sepsis.

**Figure 7:**
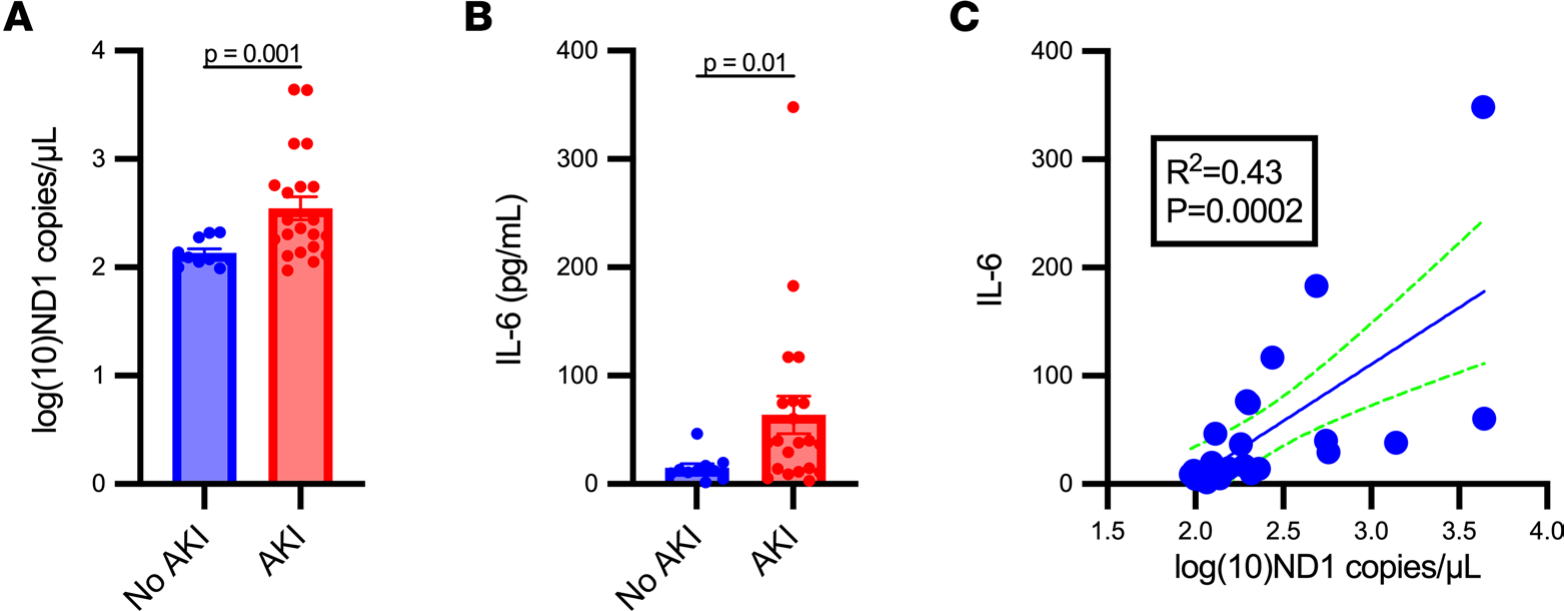
Circulating cell-free mtDNA concentrations are increased in individuals with sepsis-associated AKI compared with those with sepsis without AKI and correlate with plasma IL-6. mtDNA was quantified by droplet digital PCR targeting a 69-bp target sequence within the NADH dehydrogenase 1 (ND1) gene in plasma from a cohort of individuals with sepsis with and without acute kidney injury (AKI). (**A**) Plasma ND1 concentrations were increased significantly in individuals who developed AKI within 48 hours of ICU admission (AKI) compared with those who did not (no AKI). (**B**) IL-6 concentrations in septic individuals with AKI were significantly increased compared with those without AKI. Significant differences between groups were determined by unpaired, 2-tailed *t* tests comparing the group means ± SEM. (**C**) Simple linear regression of plasma ND1 with IL-6 concentrations in individuals with sepsis showed a positive and significant correlation. *n* = 31 patients.

We compared CCF-mtDNA concentrations in plasma from critically ill individuals with S-AKI compared to those with sepsis without AKI, as well as the association between plasma mtDNA concentrations and IL-6. Previously, we validated a human mtDNA primer/probe in the ND1 region that avoids NUMT regions in the nuclear genome and targets a small target sequence of 69 bp (30) (Table 1). In the VA San Diego Healthcare System biorepository, we identified 31 critically ill patients with sepsis at time of ICU admission in which to determine concentrations of ND1 and IL-6. Seventeen patients met criteria for AKI within 48 hours and 14 did not. Demographic data are shown in Table 3. One patient in the “no AKI” group died during hospitalization and all patients in the “AKI” group survived to discharge. The concentrations of ND1 and IL-6 were significantly increased in septic patients who developed AKI compared with those who did not (Figure 7, A and B). A simple linear regression of ND1 versus IL-6 concentrations from all septic individuals showed a positive and significant correlation (Figure 7C).

## Discussion

Investigations into mtDNA as a biomarker or modifiable mediator of sepsis-associated organ damage have been limited by a lack of characterization of CCF-mtDNA. In this study, we utilized NGS to characterize the size and sequence of CCF-mtDNA after CLP in mice to design optimal target sequences for quantification by ddPCR. In addition, a comparison of plasma and tissue mtDNA SNPs was performed to infer the tissue origin of circulating mtDNA fragments. Plasma mtDNA concentration determined by ddPCR showed a significant increase in CLP versus sham mice, which correlated temporally with increased levels of inflammatory cytokines. Most mice had plasma mtDNA fragments with SNPs that were consistent with a kidney source, and there was a greater perturbation of metabolism in the kidney compared with the heart, liver, and lung in CLP mice. We also showed that the kidney had more SNPs after CLP compared with other organs, and that kidney mtDNA contributes directly to mtDNA release from DCs. Kidney mtDNA also led to IL-6 release that was mitigated by TLR9 inhibition. Finally, we found plasma mtDNA concentrations are increased in patients with S-AKI compared with those with sepsis without AKI, with increased plasma mtDNA correlating significantly with plasma IL-6. Taken together, our results suggest that mtDNA released from the kidney contributes directly to systemic IL-6 release in sepsis.

We believe this study adds new methodological insights to approaches that may improve quantification of CCF-mtDNA. We identified a probe for mtDNA quantification by ddPCR in mice that targets a small fragment size of 93 bp that is highly unlikely to amplify NUMTs. The most utilized method of CCF-mtDNA quantification in published reports is via qPCR for one of the 37 mitochondrial genes (27). There is wide variability in the size and sequence of qPCR probes utilized in published studies, and reports of plasma levels of mtDNA and their relationship to disease have been inconsistent. Our data showing a high degree of fragmentation of CCF-mtDNA after injury suggest that probes targeting larger sequences, for example 150–200 bp, would fail to quantify the high percentage of these smaller fragments. Supporting this notion, we detected a significantly higher copy number with our CytB probe targeting a 93-bp sequence compared with ND1 probes targeting sequences of 106 and 126 bp.

Determining the tissue origin of CCF-mtDNA is challenging, as many cell types are known to release mtDNA in response to stress, and mtDNA sequence variation has yet to be specifically linked to any cell or tissue origin. However, human autopsy studies have revealed that certain mtDNA mutations develop spontaneously in specific tissues, particular with aging (34). Zhang et al. showed that a common SNP found in the kidney was also found at increased levels in plasma from a critically ill patient with AKI (35), suggesting these mutated plasma mtDNA fragments may have been of kidney origin. Our findings support this conclusion, as we found that most mice subjected to CLP had at least 1 plasma mtDNA SNP that was shared with the kidney, but not found in the liver, heart, buffy coat, or lung.

Interestingly, all mice had several SNPs in plasma that did not correspond to any of the tissues we investigated. CCF-mtDNA is highly vulnerable to oxidative damage, so it is possible that some of these SNPs develop while in the circulation. Other tissues not included in our investigation, such as brain and muscle, may also contribute. It is also important to note that there are several kidney mtDNA SNPs that are not found in the plasma. Non-tissue sources, such as leukocytes and platelets, may explain these findings, as these cells are known to expel mtDNA as part of the innate immune response (32). However, we found that buffy coat, used as a leukocyte control, did not contain any SNPs that met our criteria. Additionally, the overall percentage of mutated mtDNA is low (1%–2%) within organs and would be predicted to decrease even further when combined with other sources. Overall, our data suggest that CCF-mtDNA is likely of multiple cell and tissue sources, including the kidney in sepsis. Importantly, we also show that kidney mtDNA causes mtDNA release from DCs, suggesting kidney mitochondrial damage and mtDNA release also promote increased CCF-mtDNA concentrations during sepsis by promoting mtDNA release from other cells.

Our findings of impaired FAO and phospholipid metabolism in the kidney after CLP are consistent with previous results from Johannsen et al. who showed that changes in these 2 pathways correlated with increased plasma mtDNA concentrations in individuals with sepsis (31). Phospholipids are key components of cell membranes, including the mitochondrial membranes; thus, an accumulation of phospholipids could also be reflective of membrane damage. The role of phospholipid metabolism in S-AKI was outside the scope of the present study but warrants further investigation based on our results. FAO metabolites, such as medium- and long-chain acylcarnitines, were increased more significantly than other metabolites, and increased levels of these acylcarnitines are potential biomarkers of mitochondrial dysfunction (36). We also found that FAO metabolites, and the total metabolome, were more significantly altered in the kidney compared to the heart, liver, and lung. These findings support the notion that the kidney is a major site of mitochondrial dysfunction that leads to mtDNA damage and release during sepsis. This conclusion is supported further by our results showing significantly more kidney mtDNA SNPs after CLP compared with other organs, which may be due to increased mtDNA damage.

Tsuji et al. showed previously that CCF-mtDNA contributes directly to the development of AKI after CLP (26). We now show that the kidney cells likely contribute directly to plasma mtDNA accumulation after CLP, and kidney mtDNA may promote inflammation systemically by stimulating immune cells to release IL-6 via TLR9. Importantly, IL-6 concentrations have been shown to predict death due to CLP in mice (33), and IL-6 is perhaps the most-established mediator of remote organ injuries due to AKI in both preclinical and clinical studies (5, 8). IL-6 inhibition has shown promising results in other systemic inflammatory diseases, such as COVID-19 (37), yet the downstream consequences of IL-6 inhibition are relatively unknown and these treatments may not be beneficial in all patients. Therapies targeting mitochondrial integrity to improve kidney function during injury are ongoing (38), and our results suggest these treatments, or those that prevent mtDAMP release or remove CCF-mtDNA, may be beneficial in preventing IL-6–mediated inflammation in remote organs. A major strength of our study is that we provide a rationale for translational investigations focused on these therapeutic approaches based on the positive and significant correlation between mtDNA and IL-6 found in plasma from septic individuals. Jansen et al. did show somewhat conflicting results in a prior study where urine mtDNA was found to be associated with the systemic inflammatory response in S-AKI, but plasma mtDNA was not (25). This discrepancy may be explained by the timing of plasma mtDNA quantification, as we show increased levels of mtDNA develop early after injury, corresponding to cytokine release, and may return to normal by the time AKI is recognized clinically.

Our results also have important limitations to consider. First, plasma volume requirements for DNA recovery prevented us from being able to assess plasma mtDNA SNPs before and after CLP in individual mice. This would have been useful in determining the source of mtDNA fragments released after injury. Second, we are unable to determine exactly which cells in the kidney or other tissues contributed to the SNPs we identified. We perfused the kidney to clear resident leukocytes and other non-kidney cells at the time of harvest, but it is possible they were still a source. Third, our clinical data are limited by a relatively small cohort of 31 primarily male patients with sepsis with limited racial diversity. We also used male mice based on pilot studies where mtDNA release from males was significantly increased compared with females after CLP. Therefore, larger studies of more diverse populations, as well as those focused on sex differences in mtDNA release after CLP are warranted. These studies are especially important given mtDNA is exclusively maternally inherited, and significant racial disparities in sepsis outcomes exist (39). Finally, plasma mtDNA is known to exist in a free state or within extracellular vesicles (40), and it is unclear which pool is of pathophysiological importance. Investigations into free versus extracellular vesicle–bound mtDNA was outside the scope of this study but warrants future investigation.

In conclusion, we used NGS to characterize CCF-mtDNA after CLP to develop optimal methods to quantify mtDNA with high sensitivity and specificity in mice via ddPCR. We accounted for mtDNA fragmentation by targeting gene sequences within small mtDNA fragments and avoiding amplification of NUMTs. We found that concurrent sepsis and AKI are associated with significant elevations of plasma mtDNA concentrations. We also present evidence that kidney mtDNA may contribute to systemic inflammation in S-AKI by activating DCs to secrete mtDNA and IL-6. These findings highlight the direct role of the kidney in inflammatory crosstalk during sepsis and identify kidney mitochondrial health as a target for novel treatments focused on preventing the remote organ consequences of S-AKI. Future studies are needed to determine whether therapies preventing release of mtDNA fragments into circulation, or enhancing their clearance, may influence clinical outcomes in S-AKI.

## Methods

### Sex as a biological variable.

The animal model utilized male mice only based on pilot studies where mtDNA release from males was significantly increased compared with females after CLP. The human study recruited women; however, only 1 woman was enrolled as compared with 30 men. Given this, no conclusions can be made regarding the potential for sex as a biological variable as the source of kidney mtDNA.

### Animals and surgical procedures.

Eight- to 12-week-old C57BL/6J (Jackson Laboratory, strain 000664) male mice were randomized to CLP or sham operation. CLP was performed as described previously (10). Specifically, the cecum was ligated at 50% of its length and then punctured once with a 25-gauge sterile needle. Sham mice underwent laparotomy without any manipulation of the cecum. All mice were given 1 mg/kg of buprenorphine via intraperitoneal injection for pain at the end of surgery and again at 6 hours postoperatively. Mice were also given 1 mL of warmed sterile saline subcutaneously for resuscitation at the same time points.

### Human samples.

Plasma samples were obtained from a biorepository of 104 adults (≥18 years old) who were admitted to the ICU of the VA San Diego Healthcare System from January 2016 through December 2017. Sepsis was defined by the Third International Consensus Definitions for Sepsis and Septic Shock (Sepsis-3) (2). The presence of additional organ failures, such as heart failure or liver failure, was determined by chart review. Blood samples were collected from patients with sepsis at the time of ICU admission to generate platelet-poor plasma as described previously (41) prior to being stored at –80°C.

AKI was defined by the Kidney Disease: Improving Global Outcome (KDIGO) criteria: an increase in serum creatinine of greater than or equal to 0.3 mg/dL within 48 hours or a 50% increase from a baseline serum creatinine (42). Two groups were identified based on the presence or absence of AKI within 48 hours of admission and designated as “AKI” or “no AKI,” respectively. We chose the 48-hour window based on the rationale that mtDNA increases early after an injurious insult to the kidney, yet creatinine may not increase until 1–2 days after injury.

### Plasma creatinine and cytokine measurements.

Plasma creatinine concentration at 24 hours after CLP or sham operation was used as a marker of kidney injury, and determined by liquid chromatography with tandem mass spectrometry (LC-MS/MS) as described previously (43). Plasma and supernatant concentrations of IFN-γ, IL-10, IL-12p70, IL-1β, IL-2, IL-4, IL-5, IL-6, KC/GRO, and TNF-α were determined utilizing a V-PLEX Proinflammatory Panel 1 Mouse Kit (Meso Scale Diagnostics, K15048D) on a Meso Sector S600. IL-6 in plasma and cell supernatant collected after mtDNA experiments were determined by ELISA using the mouse IL-6 Quantikine ELISA kit (R&D Systems, M6000B) on a precoated 96-well plate. Multiplex and ELISA studies were performed as per the manufacturer’s instructions with all samples run in duplicate. IL-6 was determined in human samples via the Cobas e411 analyzer (Roche Diagnostics) per the manufacturer’s instructions.

### Library preparation and high-throughput sequencing.

Library preparation and sequencing were performed as described previously (30). Purified DNA was used to generate sequencing libraries using the KAPA Hyper Prep Kit (KAPA Biosystems) as published previously (35). Sequencing was then performed using the HiSeq 4000 sequencing system (Illumina).

### Alignment, size distribution, and heteroplasmy of mtDNA.

Sequenced reads from each sample were aligned to mouse genome mm10 with the bwa mem algorithm (https://github.com/lh3/bwa). Reads aligned to mtDNA were extracted into smaller bam files, and variants with respect to the reference mitochondrial sequence were called using the algorithm bcftools mpileup (44) subject to a minimal read mapping quality threshold of *–Q 30*, and a minimum base quality threshold of *–Q 20*. Reads with primary alignments to the nuclear genome were filtered out to avoid alignment of NUMTs. Read length was determined as the distance (+1) of the first and last aligned bases of the paired-end read. Only SNPs were reported in the variant call vcf files. SNPs from all samples taken from the same animal were summarized in 1 Microsoft Excel spreadsheet, in which each sheet corresponds to a different source tissue of DNA (plasma, heart, kidney, liver, lung, etc.). This was done using a custom R script, which reads the appropriate vcf files and writes an Excel spreadsheet as the result. Heteroplasmy sites were then identified via the following criteria as described previously in human samples (35): sequencing coverage greater than 400, minor allele frequency of 1% or greater, and minor allele frequency no less than 0.6% for both strands and not significantly different between strands. Sequencing data were uploaded to a shared server (BioProject) and may be accessed online (http://www.ncbi.nlm.nih.gov/bioproject/899505).

### Determining potential NUMT sequences.

The mouse mitochondrial genome sequence was converted into fragments of 101 bp in length, with the central nucleotide determining the position of the fragment to make 16,569 distinct fragments, with every pair of consecutive fragments having an overlap of 100 bp. The minimum Hamming distance was defined as the minimum number of mismatches between each fragment (or its reverse complement) and the best match within the entire nuclear genome using blastn in the ungapped mode (45).

### Plasma mtDNA quantification.

Plasma mtDNA concentrations were determined by ddPCR targeting the sequences shown in Table 1 as previously described (30). The droplets from each sample were read on a QX200 droplet reader machine (Bio-Rad Laboratories) and analyzed individually using QuantSoft version 1.7.4 software. PCR-positive and PCR-negative droplets were counted to provide absolute quantification of target mtDNA sequences in digital form. The fraction of positive droplets in a sample was used to determine the concentration of the target sequence in copies/μL, then multiplied by 20 to account for the dilution factor.

### Metabolite extraction and LC-MS/MS analysis.

Kidney, heart, liver, and lung samples were harvested 4 hours after CLP. Metabolite extraction was performed as described previously (7). LC-MS/MS analysis was conducted using a UHPLC system (LC-20A) coupled to a Qtrap 6500 hybrid triple quadrupole mass spectrometer (SCIEX) operated in both negative (ESI–) and positive (ESI+) modes (7). A broad-spectrum, targeted metabolomics method was utilized to evaluate a total of 483 metabolites. The metabolites evaluated cover a broad range of chemical classes that are known to be essential in metabolism, and all were confirmed by authentic standards.

### Treatment with mtDNA and mtDAMPs.

Mitochondria were isolated from mouse kidney using the Mitochondria Isolation Kit (MilliporeSigma, MITOISO1) and kidney mtDNA was isolated using the Mitochondrial DNA Isolation Kit (BioVision, K280-50) according to the manufacturers’ instructions. Kidney mtDNA was then ultrasonicated to shear it into smaller fragments, as we show CCF-mtDNA is highly fragmented. For in vitro studies, mouse bone marrow cells were harvested from C57BL/6J wild-type mice and allowed to mature for a total of 10 days to achieve bone marrow–derived DCs. Bone marrow cells were cultured in DC Base media (RPMI, 10% FBS, 2 mM L-glutamine, 1 mM NEAA, 10 mM HEPES, 55 μM β-mercaptoethanol, and 1× Pen-Strep) supplemented with 20 ng/mL GM-CSF (Peprotech). On day 7 of differentiation, the cells were split into a 12-well dish and cultured in 1 mL of GM-CSF–supplemented DC Base media. On day 10, cells were treated with CpG (1 μM) or kidney mtDNA (10 μg/mL) with or without a TLR9 inhibitor (5 μM) (InvivoGen, ODN 2088) for 1 hour prior to treatment. For in vivo experiments, a kidney mtDAMP solution was prepared to achieve adequate concentrations of mtDNA that are consistent with those found in critical illness, and the kidney mtDAMP solution was prepared as we described previously (7, 15). In these experiments, mice were sedated with inhaled isoflurane anesthesia prior to intraperitoneal injection of 50 μg/mL mtDAMP solution in 1 mL with and without 1 μg/μL of the TLR9 inhibitor ODN 2088 or 1 mL control solution (normal saline), with plasma collected as described above at 4 hours after administration.

### Statistics.

For metabolomic data analysis, peak integration was performed in MultiQuant 3.0 (SCIEX) with manual inspection and adjustment based on the standards. After peak vetting, data were exported to Excel files and data quality was checked again. The missing values were replaced by the *k*-nearest neighbors (KNN) method. Data were log_2_ transformed and autoscaled prior to statistical analysis. Metabolomic analysis, including PLS-DA and volcano plot analysis, was performed in Metaboanalyst 4.0 (https://www.metaboanalyst.ca/). The PLS-DA model was cross-validated by the LOOCV method. A VIP score of greater than 1.5 in the PLS-DA model was considered statistically significant. For volcano plot analysis, the fold-change threshold was set as 1.2 and the FDR-adjusted *P* value was set as less than 0.05. Pathway analysis was performed in Python 3.7.4 using our in-house database. The impact score of a pathway was calculated as the sum of the VIP scores for the significant metabolites normalized by the sum of VIP scores of all metabolites in that pathway. Between-group comparisons were analyzed by unpaired *t* tests or 1-way ANOVA with post hoc analysis with mtDNA data logarithmically transformed to reduce skewness and normalize data. Analyses were performed using Prism (GraphPad). Unless stated otherwise, results are presented as group means ± SEM, with *P* less than 0.05 used to determine significance.

### Study approval.

All human studies were approved by the Veterans Affairs San Diego Health System Institutional Review Board (IRB, H150104), and written informed consent was obtained prior to blood draws. Murine experiments were performed ethically in accordance with the NIH *Guide for the Care and Use of Laboratory Animals* (National Academies Press, 2011) and were approved by the Veterans Affairs San Diego Health System Institutional Animal Care and Use Committee (A18-004 and A13-026).

### Data availability.

Sequencing files can be found at http://www.ncbi.nlm.nih.gov/bioproject/899505 Values for all data points in graphs are reported in the Supporting Data Values file. The custom R script is provided in the supplemental material.

## Author contributions

MLH designed the study, performed experiments, analyzed data, interpreted results, and drafted the manuscript. AJK performed experiments, analyzed data, interpreted results, and drafted the manuscript. AS, JW, NS, MLR, AGS, KU, KL, RS, HG, KE, LAB, KJ, and XL performed experiments, analyzed data, and interpreted results. GSS, AGM, AGS, MMF, RGS, and VV helped design the study, analyze data, and interpret results. JHI and PS helped design the study, analyze data, interpret results, and draft the manuscript. All authors revised the manuscript and approved the final version.

## Funding support

This work is the result of NIH and Department of Veterans Affairs funding, in whole or in part, and is subject to the NIH Public Access Policy. Through acceptance of this federal funding, the NIH and Department of Veterans Affairs has been given a right to make the work publicly available in PubMed Central.

- Department of Veterans Affairs grants BX004338 (to MLH), I01BX003688 (to MMF), and BX002175 (to PS).
- NIH grants R01DK107852 (to PS), R01DK141973 (to PS), R01AR069876 (to GSS), K24 DK110427 (to JHI), F31AG062099 (to AGS), R01DK132690 (to VV), RF1AG061296 (to VV), T32 HL166127 (to AJK), T32CA009370-39 (to AGM), and R01DK136756 (to MLH).
- NIH P30 DK079337 and P54 DK137307 (to the University of Alabama Birmingham–UCSD O’Brien Center for AKI Research).
- NIH grant P30AI036214 (to the UCSD IGM Genomics Center, the Genomics and Sequencing Core of the San Diego Center for AIDS Research).
- The Audrey Geisel Chair in Biomedical Sciences and the NOMIS Foundation (to GSS).
- American Thoracic Society Research Foundation Unrestricted Critical Care Grant (to MLH).
- Tobacco-Related Disease Research Program grant T34IP8073 (to MMF).
- CRI Irvington postdoctoral fellowship CRI4122 (to AGM).

## Supplementary Material

Supplemental data

Supporting data values

## Figures and Tables

**Table 1:**
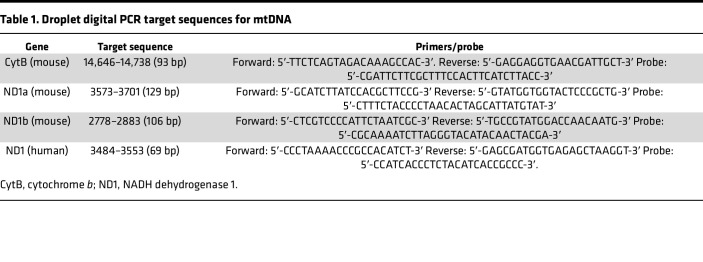
Droplet digital PCR target sequences for mtDNA

**Table 2:**
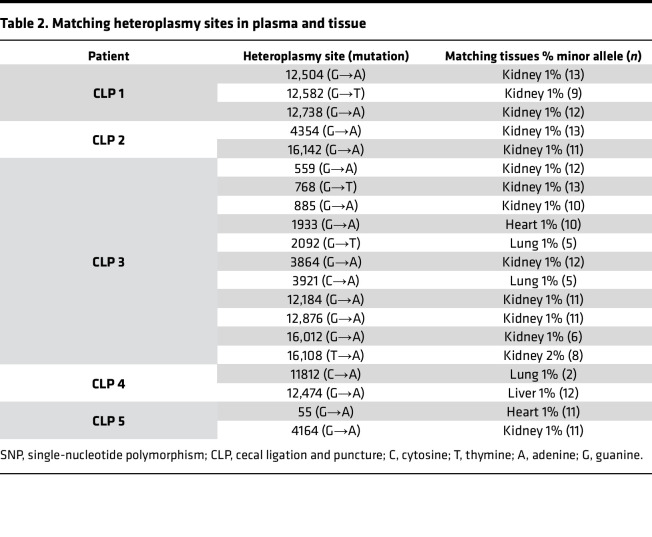
Matching heteroplasmy sites in plasma and tissue

**Table 3:**
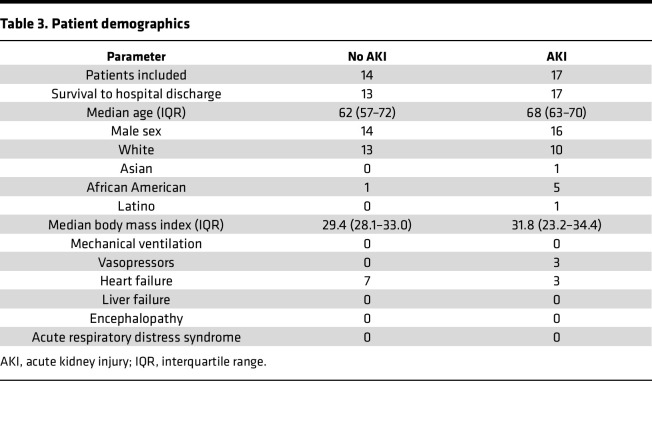
Patient demographics

## Version 1 12/08/2025

Electronic publication
