# Supplementary material for: Kidney mitochondrial DNA contributes to systemic IL-6 release in sepsis-associated acute kidney injury
Source: JCI Insight. 2025 Dec 8;10(23):e177004. doi: 10.1172/jci.insight.177004 (PMC12890523; doi:10.1172/jci.insight.177004)
Supplement: Supplemental data [file jciinsight-10-177004-s082.pdf]

SUPPLEMENTAL TABLE 1

SUBJECT	HETEROPLASMY SITE (MUTATION)	MATCHING TISSUES % MINOR ALLELE (N)
CLP #1	15951 (G->A)	KIDNEY 1% (13)
		HEART 1% (6)
		LIVER 2% (7)
	12521 (G->A)	KIDNEY 1% (11)
		HEART 2% (14)
CLP #2	11232 (G->T)	KIDNEY 2% (15)
		HEART 1% (13)
CLP #3	12219 (G->A)	KIDNEY 1% (10)
		LUNG 1% (4)
	14383 (G->A)	KIDNEY 1% (11)
		LUNG 1% (4)
	3871 (G->A)	KIDNEY 1% (10)
		HEART 1% (10)
	6132 (G->A)	KIDNEY 1% (10)
		HEART 1% (10)
		LIVER 1% (12)
CLP #4	12526 (G->A)	LUNG 1% (5)
		HEART 1% (10)
		KIDNEY 1% (10)
	16133 (T-> A)	HEART 1% (8)
		KIDNEY 7% (39)
		LIVER 5% (26)
	16108 (T->A)	KIDNEY 3% (12)
		LIVER 3% (14)
CLP #5	386 (G->T)	HEART 9% (86)
		KIDNEY 13% (125)
		LIVER 10% (91)

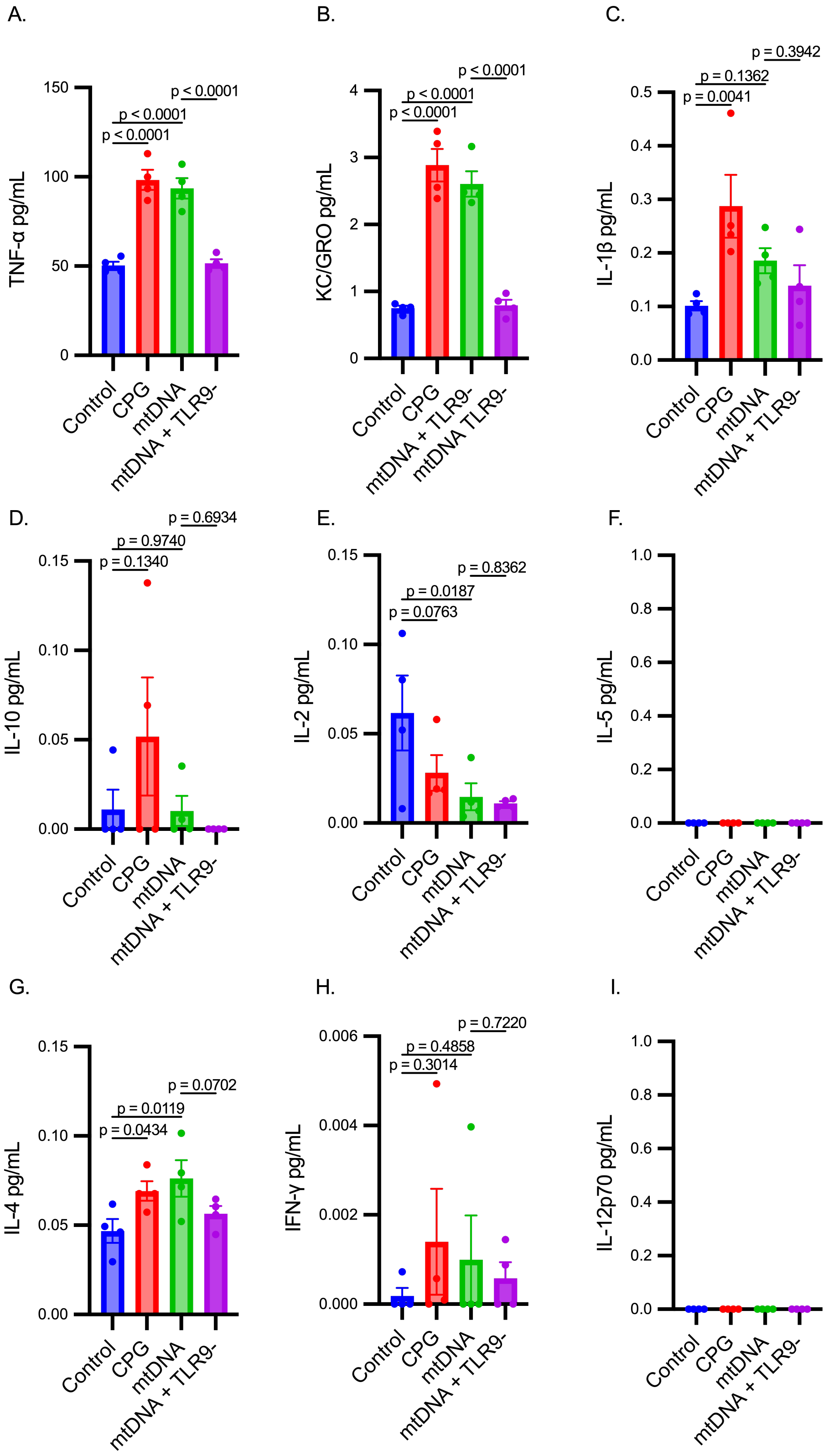
A.
TNF-α pg/mL
p < 0.0001
p < 0.0001
p < 0.0001
Control
CPG
mtDNA
mtDNA + TLR9-
B.
KC/GRO pg/mL
p < 0.0001
p < 0.0001
p < 0.0001
Control
CPG
mtDNA + TLR9-
mtDNA TLR9-
C.
IL-1β pg/mL
p = 0.3942
p = 0.1362
p = 0.0041
Control
CPG
mtDNA
mtDNA + TLR9-
D.
IL-10 pg/mL
p = 0.6934
p = 0.9740
p = 0.1340
E.
IL-2 pg/mL
p = 0.8362
p = 0.0187
p = 0.0763
F.
IL-5 pg/mL
G.
IL-4 pg/mL
p = 0.0702
p = 0.0119
p = 0.0434
H.
IFN-γ pg/mL
p = 0.7220
p = 0.4858
p = 0.3014
I.
IL-12p70 pg/mL

SUPPLEMENTAL FIGURE 1 Cytokine levels after CPG and mtDNA administration with and without TLR9 inhibition *in vitro*. Dendritic cells (DCs) isolated from mouse bone marrow were treated with CpG (1μM) as a positive control or kidney mtDNA (10 μg/mL) and incubated for 4 hrs prior to quantification of cytokines in the culture supernatant. Additional cells were treated with TLR9 inhibitor. TNF- α (A), KC/GRO (B), and IL-4 (G) levels significantly increased with CPG and mtDNA exposure. However, only TNF- α and KC/GRO levels decreased with TLR9 inhibition. Significant differences between groups were determined by one way ANOVA with post-hoc analysis. N=4/group. There was no expression of IL-5 or IL-12p70 in any group.

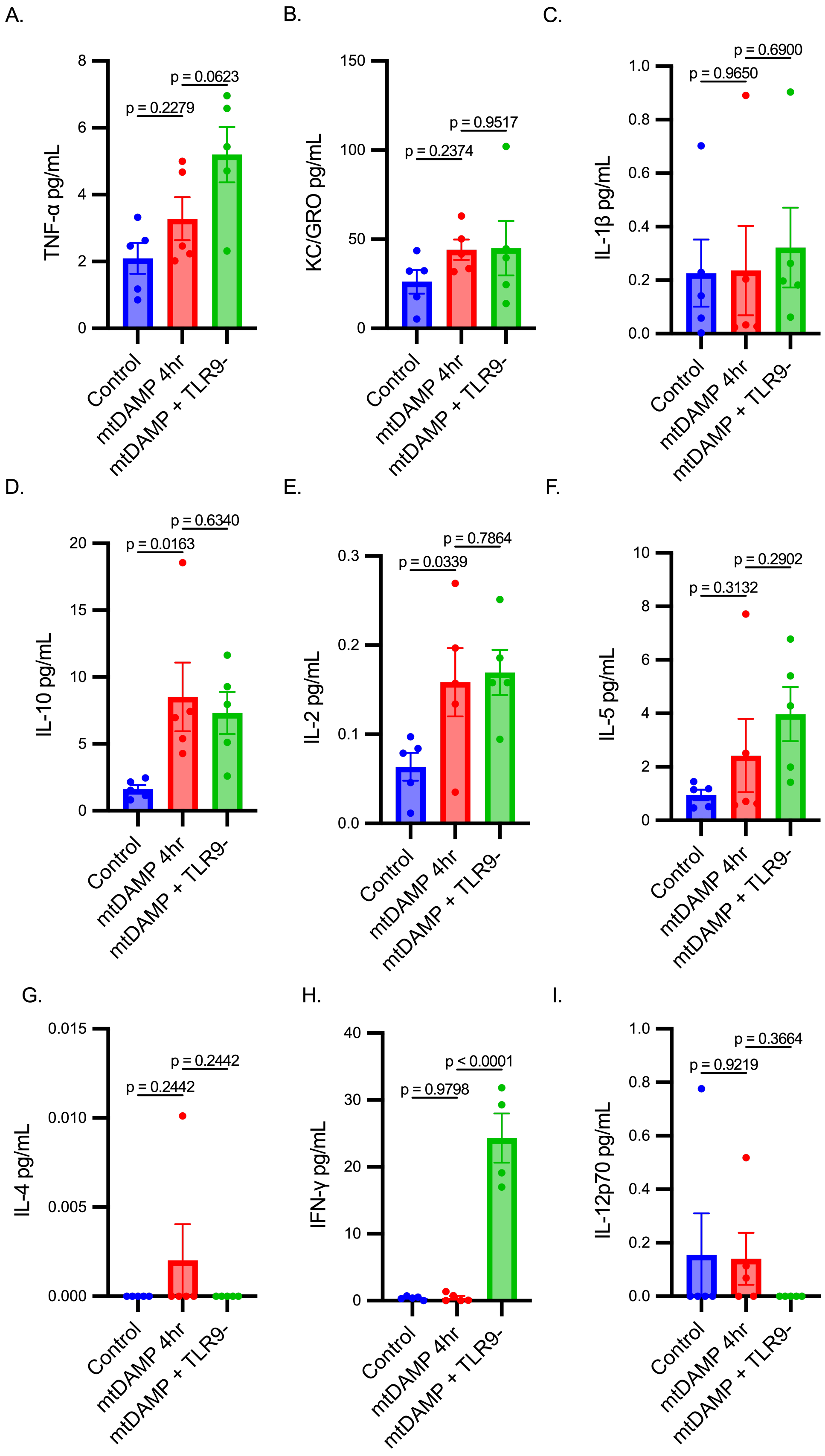
A.
TNF-α pg/mL
p = 0.2279
p = 0.0623
B.
KC/GRO pg/mL
p = 0.2374
p = 0.9517
C.
IL-1β pg/mL
p = 0.9650
p = 0.6900
D.
IL-10 pg/mL
p = 0.0163
p = 0.6340
E.
IL-2 pg/mL
p = 0.0339
p = 0.7864
F.
IL-5 pg/mL
p = 0.3132
p = 0.2902
G.
IL-4 pg/mL
p = 0.2442
p = 0.2442
H.
IFN-γ pg/mL
p = 0.9798
p < 0.0001
I.
IL-12p70 pg/mL
p = 0.9219
p = 0.3664
Control
mtDAMP 4hr
mtDAMP + TLR9-

SUPPLEMENTAL FIGURE 2 Cytokine levels after mtDNA administration at 4 hrs with and without TLR9 inhibition *in vivo*. Plasma cytokine levels were evaluated in mice at 4 hrs following subcutaneous injection of mtDAMPs with and without TLR9 inhibition vs normal saline. IL-10 (D) and IL-2 (E) increased significantly with mtDAMP treatment. Significant differences between groups were determined by one way ANOVA with post-hoc analysis. N=5/group.

Custom R Script

```
#R script to separate Plasma Heart Kidney Liver Lung
library(vcfR)
library(openxlsx)

msamples<-list()
msamples[[1]]<-c("43_S93","1_S30","2_S31","3_S32","4_S33")
msamples[[2]]<-c("44_S94","5_S34","6_S35","7_S36","8_S37")
msamples[[3]]<-c("45_S95","9_S38","10_S39","11_S40","12_S41")
msamples[[4]]<-c("46_S96","13_S42","15_S44","14_S43","16_S45")
#this order is such that PLASMA HEART KIDNEY LIVER LUNG is
preserved
msamples[[5]]<-c("47_S97","17_S46","18_S47","19_S48","20_S49")

#now combined controls
msamples[[6]]<-
c("48_S98","49_S99","50_S100","51_S101","52_S102") #these are
all PLASMA controls


for (mouse in 1:6) {
samples<-msamples[[mouse]]
ns<-length(samples)


vcf.list<-list()
for (sample in samples) vcf.list[[sample]]<-
read.vcfR(paste0(sample,".vcf"))
#they have different lengths, so create a data frame with 16299
lines, one per nt
nMT<-16299


res<-list()

for(s in samples) {
   cat(s,"\n")
   vcf<-vcf.list[[s]]

   pos<-as.numeric(vcf@fix[,2])
   df<-
data.frame(POS=1:nMT,REF=rep("",nMT),ALT=rep("",nMT),DP=rep(0,nM
T),AD=rep("",nMT),GT=rep("",nMT),PREF=rep(1,nMT),REFFRAC=rep(1,n
MT))

   G<-vcf@gt
```

```
    FX<-vcf@fix

    GT<-G[,2]
    df[pos,6]<-GT

    REF<-FX[,4]
    df[pos,2]<-REF

    ALT<-FX[,5]
    df[pos,3]<-ALT

    depth<-FX[,8]
    depth<-sapply(depth,function(x) strsplit(x,";"))
    depth<-sapply(depth,function(x) strsplit(x[1],"="))
    depth<-sapply(depth,function(x) as.numeric(x[2]))
    df[pos,4]<-depth

    ad<-FX[,8]
    ad<-sapply(ad,function(x) strsplit(x,";"))
    ad<-sapply(ad,function(x) strsplit(x[2],"="))
    ad<-sapply(ad,function(x) x[2])
    df[pos,5]<-ad

    #probability that the base is reference
    GP_list<-strsplit(GT,",")
    GP_p<-lapply(GP_list,function(x) 10^(-as.numeric(x)/10))
    #normalize probabilities
    GP_p<-lapply(GP_p,function(x) x/sum(x))
    GP_p0<-sapply(GP_p,function(x) x[1])
    df[pos,7]<-GP_p0

    #what is the fraction of mutated alleles?
    ad<-lapply(ad,function(x) strsplit(x,","))
    ad<-lapply(ad, function(x) x[[1]])
    ad<-lapply(ad,as.numeric)
    REFFRAC<-sapply(ad,function(x) x[1]/max(1,sum(x)))
    df[pos,8]<-REFFRAC

    res[[s]]<-df
}
write.xlsx(res,file=paste0("M",mouse,".xlsx"),overwrite=TRUE)

#the code below is only for mouse = 1:5, who have Heart
if (mouse %in% c(1:5)) {
    #same ALT in plasma and kidney, no ALT in Heart
```

```
   s<-(1-res[[1]]$REFFRAC)*(1-
res[[3]]$REFFRAC)*ifelse(res[[2]]$REFFRAC==1,1,0)*ifelse(res[[1]
]$ALT==res[[4]]$ALT,1,0)
   o<-order(s,decreasing=TRUE)
   #report positions

   ngood<-sum(s>0)
   ogood<-o[1:ngood]
   #create sorted output by interest
   ures<-res
   for (sample in samples) {
      ures[[sample]]<-res[[sample]][ogood,]
   }
   write.xlsx(ures,file=paste0("M",mouse,".candidate.pos.xlsx"),
overwrite=TRUE)
   }
}
```
